# Tripartite Evolutionary Game and Simulation Analysis of Healthcare Fraud Supervision under the Government Reward and Punishment Mechanism

**DOI:** 10.3390/healthcare11131972

**Published:** 2023-07-07

**Authors:** Change Zhu, Lulin Zhou, Xinjie Zhang, Christine A. Walsh

**Affiliations:** 1 Department of Management, Jiangsu University, 301 Xuefu Road, Jingkou District, Zhenjiang 212001, China; 2 Faculty of Social Work, University of Calgary, 2500 University Drive NW, Calgary, AB T2N 1N4, Canada

**Keywords:** reward and punishment mechanism, supervision of healthcare fraud, evolutionary game, simulation analysis, Matlab, China

## Abstract

This study aims to provide useful insights for the Chinese government in dealing with healthcare fraud by creating an evolutionary game model that involves hospitals, third-party entities, and the government based on the government reward and punishment mechanism. This paper analyzes the evolutionary stability of each participant’s strategy choice, discusses the influence of each element on the tripartite strategy choice, and further analyzes the stability of the equilibrium point in the tripartite game system. The results show that (1) the government increasing fines on hospitals is conducive to compliant hospital operations, and the incentive mechanism has little effect on such operations; (2) the lack of an incentive mechanism for third parties results in false investigations by third parties; and (3) rewards from higher levels of government promote strict supervision by local governments, but that the high cost of supervision and rewards for hospitals inhibits the probability of strict supervision. Finally, Matlab 2020a is used for simulation analysis to provide a reference for the government to improve the supervision of healthcare fraud.

## 1. Introduction

Healthcare fraud or abuse refers to the intentional deception or an unintentional mistake made by a person or entity to deceive the healthcare system to receive unlawful benefits or payments [1]. In recent years, healthcare fraud by various interested parties is commonplace [2], which directly results in the deficit of the national insurance fund [3] and an increase in insured patients’ economic burden, destroying the sustainable development of healthcare and affecting the public’s utilization of healthcare services [4]. Healthcare fraud is a worldwide problem [5,6,7] and commonly occurs in the US, China, and Indonesia [8], among other countries—especially in China. According to the National Medical Insurance Bureau in China, in 2021, healthcare administrative departments dealt with 414,000 illegal institutions, including 4181 medical insurance service agreements, 7088 administrative penalties, and 404 judicial authorities. A total of CNY 23.418 billion in medical insurance funds was recovered throughout the year (data source: http://www.nhsa.gov.cn/, accessed on 20 January 2023), which had a serious impact on socioeconomic development and the utilization of healthcare [9]. Accordingly, it is imperative for China to investigate the essential problem behind healthcare fraud.

The provision, usage, and management of healthcare involve many sectors and stakeholders, including local governments, hospitals, and patients. Healthcare systems are very vulnerable to fraud and abuse due to the complex relationships among stakeholders [10]. Compared to local governments and patients, hospitals have inherent advantages in terms of information resources and professional medical knowledge during the provision of the health care, which makes it easier for hospitals to manipulate medical treatment, eventually resulting in healthcare fraud [8]. Thus, hospitals are usually the main fraudsters in healthcare [11]. Specifically, hospitals often bill for the healthcare services or goods that have not been rendered or for unnecessarily performed medical operations and prescribed medications [4]. Excessive diagnosis and treatment, drug use beyond the required scope, and the dispensing of drugs for insured individuals by means of decomposition or prescription change are also potential healthcare fraud behaviors [12]. The illegal behaviors mentioned above not only cause serious economic burdens on patients but also cause great waste in our medical insurance fund.

Therefore, strengthening the investigation and detection of healthcare fraud is the key to safeguarding public interests, ensuring the safety of health insurance funds, and promoting the long-term development of healthcare security [13]. Although local governments intend to promote the optimization of healthcare behavior, due to the lack of professional knowledge in the field of medical and health services, the lack of experience in event judgment, and the lack of support from the corresponding professional and technical personnel, it is difficult to ensure the actual effect of the supervision of healthcare fraud, preventing the management of frequent healthcare fraud.

In this regard, in order to ensure compliance in healthcare provision and reduce the problem of healthcare fraud, the Chinese government has started to innovate with respect to the supervision mode of healthcare fraud and introduced a third-party regulatory agency [14,15]. The government’s introduction of a third party to participate in the supervision of healthcare fraud is designed to help strengthen the professionalism, independence, accuracy, and effectiveness of the supervision. However, the third party is a for-profit institution. Driven by potential interests, there is a risk of the third party engaging in rent-seeking behavior, which could lead to the loss and waste of the nation’s medical insurance fund, placing a heavy economic burden on insured patients. Accordingly, local governments still need to supervise the investigations of the third party, as hospitals, the third party, and local governments are all players of a mutual game. Therefore, the rent-seeking behavior of the third party must be comprehensively considered in order to design a supervision mechanism for healthcare fraud. Hence, this study aims to provide useful insights for the Chinese government in dealing with healthcare fraud by creating a game model that involves hospitals, third-party entities, and the government based on the government reward and punishment mechanism. By using this model, we can analyze the different strategies chosen by each participant and identify the factors that influence their decision-making process, which will shed light on effective approaches for governing healthcare fraud and guide the government’s actions in this area.

## 2. Literature Review

### 2.1. Supervision of Healthcare Funds

At present, the problem of fraud in healthcare has attracted worldwide attention, including from scholars and governments within the past decade [16,17]. Existing research is mainly focused on the responsibility of the supervision subject [18,19], the supervision method [20,21], the supervision legal system [22], and the reasons for healthcare fraud [23,24].

#### 2.1.1. Research on the Supervision Subject

First, in regard to the supervision subject, scholars have two main points of view on who bears the responsibility for the supervision of healthcare fraud. The Organization for Economic Co-operation and Development (OECD, 2002) proposed that it was important to distinguish the responsibilities and obligations of regulatory subjects and build an organizational structure that clearly divided regulatory responsibilities [18]. Some scholars are of the opinion that the government should bear the supervision responsibilities. For instance, Gillion and Turner (2000) employing the perspective of the definition of government responsibility, offered that the government’s regulatory powers and boundaries should be clearly defined so as to establish a long-term mechanism for healthcare fraud supervision [19]. Arthur (1995) pointed out that the intervention of government departments played an important role in the operation of medical insurance funds, but the role should be moderate to avoid regulatory failure caused by complete intervention [25]. Other researchers maintain that the society and the market should be the core of supervision. For example, Ginter (2018) introduced the importance of social supervision and opined that social supervision and broadly mobilizing the public to supervise the legitimacy and rationality of fund use is a widely used form of supervision [26].

#### 2.1.2. Research on the Supervision Method

It is of great practical significance to strengthen the supervision of healthcare fraud to promote the fair and stable development of the economy and society. As for the means of supervision, research has shown that information technology played a key role in the field of healthcare fraud [20,21]. Numerous researchers have used machine learning to detect medical fraud with positive outcomes [27]. Specifically, it was proposed that the use of artificial intelligence and blockchain technology to detect the security of medical insurance funds can improve regulatory efficiency and that the application of big data technology can significantly reduce the problem of healthcare fraud through case studies [28,29]. Additionally, data-mining techniques may improve the accuracy and efficiency detection of healthcare fraud [12].

#### 2.1.3. Research on the Supervision Legalization

In addition to the use of supervision, the legalization construction of preventing healthcare fraud should be considered [22]. However, privacy laws may be an obstacle to managing fraud on an organizational scale [30]. Hence, laws and the legal construction of healthcare fraud supervision should be put forward urgently [31]. The National Health Care Anti-Fraud Association (NHCAA) outlined several key factors to guide medical fraud laws, including the use of predictive models and treatment transparency between private insurance companies and government programs [32].

#### 2.1.4. Research on the Reason of Healthcare Fraud

Faced with so many healthcare fraud events, the reasons behind the fraud behavior must be considered. It is common knowledge that the process of healthcare provision and usage involves multiple subjects, such as healthcare management departments (the local government), healthcare providers (hospital and physician), and insured persons [33]. Therefore, it can be inferred that the fundamental reason for healthcare fraud is that multiple agents in the game system all pursue their own interests to maximize their own benefit. Moreover, the hospital, as the core of the game group, having information advantages, and thus informational asymmetry within multi-agents is the main cause of committing healthcare fraud [23,24].

### 2.2. Evolutionary Game Model

So far, the focus of game theory research is on the decisions made when subjects interact with each other. Since its first appearance in the 1950s, game theory has been widely used in the fields of economy, finance, and energy [34]. However, the traditional game theory has some defects [35,36]. First, traditional game assumes that all participants are completely rational, which is inconsistent with reality. Secondly, traditional game theory cannot dynamically describe the evolution of the strategic behavior of participants, lacks analysis and explanation on the process of reaching a stable equilibrium point, and ignores the dynamic research on the evolution process. Evolutionary game theory, originated in the 1990s, is an improvement on traditional game theory, whose limitations it aims to solve [37].

Evolutionary games, which combines game theory and dynamic evolutionary process analysis, do not require participants to be completely rational or have complete information. Also, evolutionary games emphasize dynamic equilibrium, unlike traditional game theory, which focuses on static equilibrium [38]. The evolutionary game takes multi-agents as the research objects to analyze the changes of each agent’s strategies over time and explains why and how to reach the current state.

Evolutionary game theory, first applied to the field of biological evolution [39], was then gradually applied to other fields. Nowadays, evolutionary game theory is widely used in pollution governance. For instance, the tripartite evolutionary game was used to analyze the problem of heavy metal pollution control and study the behavior selection strategies of different participants under changing parameters [40]. Moledina used evolutionary game theory to analyze the impact mechanism of government policies on soil pollution behavior [41]. Also, the tripartite evolutionary game was utilized to analyze green agro-product supply in an agricultural industrialization consortium [42]. Finally, Zhu used a tripartite evolutionary game to analyze the impact of renewable portfolio standards on the retail electricity market [43].

To sum up, the extant research on healthcare fraud is primarily based on the analysis of existing fraud circumstances, fraud detection, and countermeasures. As this body of research lacks information on the essential interest relationship behind the healthcare fraud, it is unable to fundamentally solve the problem of healthcare fraud. Also, tripartite evolutionary game has been applied to many fields [40,42], but it has not yet been applied to the governance of healthcare fraud. As mentioned above, the hospital, the third party, and the government are in a game with each other during the supervision of healthcare fraud. Also, the hospital bribed the third party in order not to be supervised, so there is rent-seeking behavior on behalf of the hospital in this game relationship. Tripartite evolutionary game is an effective method to study the dynamic changes of multi-agent strategies with bounded rationality in long-term repeated games, which is applicable to the study of rent-seeking behavior [44,45]. Hence, this study intends to apply the tripartite evolutionary game to examine the supervision of healthcare fraud. It can be inferred from the above analysis that without the constraint of a reward and punishment mechanism, the hospital and the third party have committed violations in pursuit of their own potential interests. Therefore, this study considers the government’s reward and punishment parameters in designing the mechanism to constrain the illegal operation of the hospital and the rent-seeking behavior of the third party.

In view of this circumstance, this paper considers the rent-seeking behavior between the hospital and the third party; constructs a tripartite evolutionary game model with the hospital, third-party institutions, and the local government as the main body; and analyzes the stability of each player’s strategy and the impact of each factor on strategy selection. In addition, the Lyapunov first method is used to analyze the stability of the equilibrium point of pure strategy of the replicated dynamic system, and the evolutionarily stable strategy combinations under different conditions are obtained. Finally, the simulation analysis is carried out using Matlab 2020a, which verifies the effectiveness of the model analysis under different initial conditions and proposes countermeasures and suggestions for the government to improve the regulatory mechanism of healthcare fraud according to the analysis conclusions.

## 3. Model Assumptions and Construction

**Figure 1:**
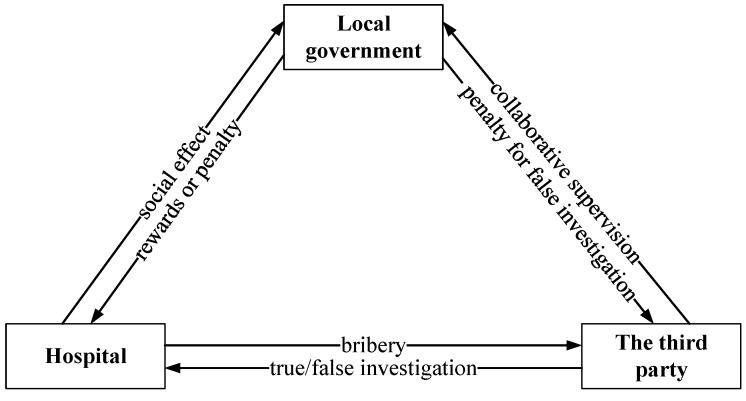
The relationship of game players.

As mentioned above, the research subjects of this paper include the hospital, the third party and the local government. In China, the local government is the main body of healthcare fraud supervision. However, due to the complexity of the relationship between multiple subjects and the professionality of healthcare supervision, single-body supervision is prone to weak supervision, so the local government normally introduces a third party to coordinate supervision. As we know, the third party has professional medical health and regulatory resources that can effectively help the government find out the violations of the hospital so as to punish the violator and rectify the wrongdoings of the hospital. In China, third-party regulatory agencies involved in healthcare fraud prevention typically have direct collaboration or employment ties with the government, operating under government supervision and guidance. This close partnership establishes a stronger connection and coordination between the third party and the government, with the government playing a crucial role in the regulatory process. Nevertheless, despite the close affiliation between the third party and the government in China, the lack of effective oversight often enables these third parties to exploit hospitals for their own financial benefit. Furthermore, the government’s practice of assigning a single third party to investigate hospitals can contribute to rent-seeking behavior. In addition, the third party in China is typically employed by the government and receives a fixed salary. As a result, there is no additional incentive for the third party to conduct thorough investigations, leading to a lower rate of true investigations. Hence, it becomes necessary for the government to monitor and supervise the conduct of the third party as well. If the third party seeks rent from the hospital and commits a false investigation, it will be fined by the local government. In this tripartite relationship, the hospital is the core subject of supervision. The government and the third party both supervise the healthcare provision of the hospital. The relationship of the game player is shown in Figure 1.

In order to build a game model and analyze the stability of the equilibrium point of each party’s strategies and the influence of each element, the following assumptions are put forward:

**Assumption 1.**  *This paper sets the hospital as participant 1, the third party as participant 2, and the local government as participant 3. All three parties are participants with limited rationality, and the strategy selection gradually evolves and stabilizes to the optimal strategy over time [46].*

**Assumption 2.**  *The strategy space of the hospital is (compliant operation; illegal operation), in which the probability* x *is used to select compliant operation, and the probability* 1−x *is used to select illegal operation,*  x∈[0,1]*. The strategy space of the third party is (true investigation; false investigation), in which the true investigation is selected with probability* y*, and the false investigation is selected with probability* 1−y*,* y∈[0,1]*. The strategy space of the local government is (strict supervision; non-supervision), in which strict supervision is selected with probability* z*, and non-supervision is selected with probability*  1−z*,* z∈[0,1].

**Assumption 3.**  *Cost. The cost of compliant operation of hospital is* $C_{h1}$*, and the cost of illegal operation is* $C_{h2}(C_{h1}>C_{h2})$*. The cost of a true investigation by the third party is* $C_{t1}$*, and the cost of a false investigation is* $C_{t2}(C_{t1}>C_{t2})$*. The cost of strict supervision of the local governments is* $C_{g}$*, and the cost of non-supervision is 0*$\left(C_{g}>0\right)$.

**Assumption 4.**  *Incomes. When a hospital operates in compliance, it gains income* $I_{h1}$*, and if it operates illegally, it gains income* $I_{h2}(I_{h2}>I_{h1})$*. When a hospital operates in compliance with the regulations, the third party can obtain income* $I_{t1}$ *through a true investigation. If a hospital operates in violation of the regulations and the third party conducts false investigation, the third party can obtain bribes from the hospital and thus the third party can obtain income* $I_{t2}(I_{t2}>I_{t1})$*. The local government gains social benefits* $I_{g1}$ *when the hospital operates in compliance and gains social benefits* $I_{g2}(I_{g1}>I_{g2})$ *when it operates in violation of regulations.*

**Assumption 5.**  *Reward and punishment mechanism. When the local government uses a strict supervision strategy, the hospital can obtain rewards from local government* $A_{h}(A_{h}<C_{h1})$* for compliant operation. If the hospital operates in violation of regulations and the third party conducts false investigation, the government will impose fines on the hospital* $P_{h}$ *and the third party* $P_{t}$*. If the local government takes a non-supervision strategy, no reward will be given. However, if the third party takes a true investigation strategy and finds that the hospital operates in violation of regulations, the government will still impose fines* $P_{h}$ *on the hospital. In addition, to encourage the local government to strictly supervise the operation of hospital, the superior government will reward the local government* $A_{g}(A_{g}<C_{g})$*. If the hospital operates in violation of regulations, and the local government and the third party adopt non-supervision and false investigation strategies, respectively, resulting in the loss of social benefits, the local government will be fined* $P_{g}$ *by the superior government. The details of the parameters are shown in* Table 1*, and the payoff matrix for the evolutionary game model is exhibited in* Table 2.

## 4. Evolutionary Game Analysis

### 4.1. Hospital Evolution Stability Strategy

Assuming that the hospital chooses the ‘compliant operation’ strategy with an expected rate of return of $E_{11}$, the ‘illegal operation’ strategy has an expected rate of return of $E_{12}$, and the average expected rate of return is $\bar{E_{1}}$ as follows:

(1) $$E_{11}=yz(I_{h1}+A_{h}-C_{h1})+y(1-z)(I_{h1}-C_{h1})+(1-y)z(I_{h1}+A_{h}-C_{h1})+(1-y)(1-z)(I_{h1}-C_{h1})$$

(2) $$E_{12}=yz(I_{h2}-C_{h2}-P_{h})+y(1-z)(I_{h2}-C_{h2}-P_{h})+(1-y)z(I_{h2}-C_{h2}-P_{h})+(1-y)(1-z)(I_{h2}-C_{h2})$$

(3) $$\bar{E_{1}}=xE_{11}+(1-x)E_{12}$$

Therefore, the replicated dynamic equation of the hospital’s ‘compliant operation’ strategy is as follows:

(4) $$F(x)=\frac{dx}{dt}=x(E_{11}-\bar{E_{1}})=x(1-x)(C_{h2}-C_{h1}+I_{h1}-I_{h2}+zA_{h}+yP_{h}+zP_{h}-yzP_{h})$$

A partial derivative of the replicated dynamic equation of the hospital’s choice of the ‘compliant operation‘ strategy can be obtained as follows:

(5) $$\frac{d(F(x))}{dx}=(1-2x)(C_{h2}-C_{h1}+I_{h1}-I_{h2}+zA_{h}+yP_{h}+zP_{h}-yzP_{h})$$

(6) $$\mathrm{We}\;\mathrm{mark}\;G(y)=C_{h2}-C_{h1}+I_{h1}-I_{h2}+zA_{h}+yP_{h}+zP_{h}-yzP_{h}$$

whose derivative is as follows: $\partial G(y)/\partial y=P_{h}(1-z)>0$.

According to the stability theorem of differential equation, the probability of hospital choosing the ‘compliant operation’ must meet the following conditions when it is in a stable state, that is, F(x)=0 and d(F(x))/dx<0. Due to ∂G(y)/∂y>0, G(y) will increase with y. It can be obtained through calculation that when $y=\frac{C_{h2}-C_{h1}+I_{h1}-I_{h2}+zA_{h}+zP_{h}}{P_{h}(z-1)}=y*$, G(y)=0, $\frac{d(F(x))}{dx}\equiv 0$, a stable state cannot be achieved; when y<y*, G(y)<0, $\frac{d(F(x))}{dx}{|}_{x=0}<0$, x=0 is an evolutionary stability strategy; and when y>y*, G(y)>0, $\frac{d(F(x))}{dx}{|}_{x=1}<0$, x=1 is an evolutionary stability strategy.

**Figure 2:**
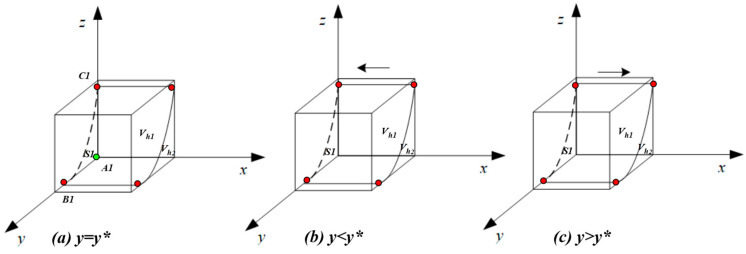
Replicated dynamic phase diagram of the hospital.

Let formula $G(y)=C_{h2}-C_{h1}+I_{h1}-I_{h2}+zA_{h}+yP_{h}+zP_{h}-yzP_{h}=0$, and from this it can be determined that $y=\frac{C_{h2}-C_{h1}+I_{h1}-I_{h2}+zA_{h}+zP_{h}}{P_{h}(z-1)}=y*$. Based on this formula, it can be calculated that $y^{*}$ will pass through point $B_{1}\;(0,\frac{C_{h1}-C_{h2}-I_{h1}+I_{h2}}{P_{h}})$ and point $C_{1}\;(\frac{C_{h1}-C_{h2}-I_{h1}+I_{h2}}{A_{h}+P_{h}},0)$. Due to $\frac{C_{h1}-C_{h2}-I_{h1}+I_{h2}}{P_{h}}>0,\frac{C_{h1}-C_{h2}-I_{h1}+I_{h2}}{A_{h}+P_{h}}>0$, an approximate image of the $y^{*}$ function can be obtained (shown in Figure 2). To be specific, subfigure (a) represents the unstable condition when $y=y^{*}$, subfigure (b) shows that x = 0 was the ESS when $y<y^{*}$, and subfigure (c) shows that x = 1 was the ESS when $y>y^{*}$.

Figure 2 shows the replicator dynamics and evolutionary stability strategies of the hospital’s choice of the ‘compliant operation’.

As shown in Figure 2, the whole space is divided into region $V_{h1}$ and region $V_{h2}$. $V_{h1}$ represents the probability that the hospital will select the ‘compliant operation’, and $V_{h2}$ represents the probability that the hospital will select the ‘illegal operation’, as follows:

The area formed by $A_{1},B_{1},C_{1}$ is marked as $S_{1}$, which is the planar area of $V_{h2}$($V_{h2}=1*S_{1}$), indicating the $V_{h2}$ will increase as $S_{1}$ increases.

It is calculated that $A_{1}B_{1}=\frac{C_{h1}-C_{h2}-I_{h1}+I_{h2}}{A_{h}+P_{h}}$, $A_{1}C_{1}=\frac{C_{h1}-C_{h2}-I_{h1}+I_{h2}}{P_{h}}$, so the larger the values of $A_{1}B_{1}$ and $A_{1}C_{1}$, the larger the values of $S_{1}$ and $V_{h2}$. Due to $V_{h1}=1-V_{h2}$, the smaller the values of $A_{1}B_{1}$ and $A_{1}C_{1}$, the larger the value of $V_{h1}$.

**Inference 1.**  *The probability of the ‘compliance operation’ of the hospital* $V_{h1}$ *is related to the government’s reward* $A_{h}$ *and the punishment for hospital* $P_{h}$*, as well as their own operating costs* $C_{h1}$*,* $C_{h2}$* and operating benefit* $I_{h1}$*,*$I_{h2}$*. Specifically, the probability of the ‘compliance operation’ of the hospital is positively related to the rewards* $A_{h}$*, the punishments from the local governments* $P_{h}$*, the incomes of ‘compliant operation’ of hospital* $I_{h1}$*, and the costs of the ‘illegal operation’* $C_{h2}$*. The probability of the ‘illegal operation’ of the hospital* $V_{h2}$ *is positively related to the incomes of the ‘illegal operation’ of the hospital* $I_{h2}$ *and the cost of the ‘compliant operation’* $C_{h1}$.

**Proof.**  The first order partial derivative of each element for $A_{1}B_{1}$ is calculated as follows:

$$\partial A_{1}B_{1}/\partial A_{h}<0,\partial A_{1}B_{1}/\partial P_{h}<0,\;\partial A_{1}B_{1}/\partial C_{h1}>0,\;\partial A_{1}B_{1}/\partial C_{h2}<0,\;\partial A_{1}B_{1}/\partial I_{h1}<0,\;\partial A_{1}B_{1}/\partial I_{h2}>0$$

Based on the positive relationship between $A_{1}B_{1}$ and $V_{h2}$, it can be concluded that

$$\partial V_{h2}/\partial A_{h}<0,\;\partial V_{h2}/\partial P_{h}<0,\;\partial V_{h2}/\partial C_{h1}>0,\;\partial V_{h2}/\partial C_{h2}<0,\;\partial V_{h2}/\partial I_{h1}<0,\;\partial V_{h2}/\partial I_{h2}>0$$

Due to $V_{h1}=1-V_{h2}$, it can be inferred that

$$\partial V_{h1}/\partial A_{h}>0,\;\partial V_{h1}/\partial P_{h}>0,\;\partial V_{h1}/\partial C_{h1}<0,\;\partial V_{h1}/\partial C_{h2}>0,\;\partial V_{h1}/\partial I_{h1}>0,\;\partial V_{h1}/\partial I_{h2}<0$$

From Inference 1, it can be seen that the local government can encourage the hospital to operate in compliance with regulations by increasing the incentives for the hospital. Similarly, for the hospital that operates in violation of regulations, the government can increase the punishment for illegal operations to drive hospital to operate in compliance with regulations. In addition, raising the cost of the illegal operation of the hospital can encourage the hospital to operate in compliance. Conversely, reducing the cost of the compliant hospital operation can compliant operations. Finally, improving the incomes of compliant hospital operation means that the hospital will tend to operate in compliance. On the contrary, reducing the revenue of the illegal hospital operations will drive the hospital to operate in compliance. Therefore, when the hospital operates in compliance, the local government will increase incentives to the hospital, which is conducive to encouraging the hospital to maintain their compliant operation strategy choice. When the hospital operates in violation of regulations, the local government will increase the fines on the hospital, which will drive hospital to choose compliant operation strategies. □

**Inference 2.**  *The probability of the ‘compliant operation’ of the hospital will increase with the increase of the probability of a ‘true investigation’ by the third party or the probability of ‘strict supervision’ by the government.*

**Proof.**  It can be determined from the strategic stability of the hospital that

When $y<\frac{C_{h2}-C_{h1}+I_{h1}-I_{h2}+zA_{h}+zP_{h}}{P_{h}(z-1)}$ or $z<-\frac{C_{h2}-C_{h1}+I_{h1}-I_{h2}+yP_{h}}{A_{h}+P_{h}(1-y)}$, G(y)<0, $\frac{d(F(x))}{dx}{|}_{x=0}<0$, x=0 will be an evolutionary stabilization strategy (the ESS point). When $y>\frac{C_{h2}-C_{h1}+I_{h1}-I_{h2}+zA_{h}+zP_{h}}{P_{h}(z-1)}$ or $z>-\frac{C_{h2}-C_{h1}+I_{h1}-I_{h2}+yP_{h}}{A_{h}+P_{h}(1-y)}$, G(y)>0, $\frac{d(F(x))}{dx}{|}_{x=1}<0$, x=1 will be an evolutionary stabilization strategy (the ESS point). In other words, the larger the values of y,z, the larger the value of x.

It can be obtained from Inference 2 that the probability of the ‘true investigation’ of the third party is conducive to the hospital adopting the stable strategy of a ‘compliant operation’. Similarly, the local government can promote the hospital to adopt the stable strategy of a ‘compliant operation’ by increasing the probability of strict supervision. Therefore, when the third party adopts a true investigation strategy and the local government adopts a strict supervision strategy, increasing the reward for the compliant operation of the hospital or increasing the fines for the illegal operation of the hospital will drive the hospital to adopt the stable strategy of ‘compliant operation’. □

### 4.2. Third Party Evolution Stability Strategy

Assuming that the third party chooses the ‘true investigation’ strategy with an expected rate of return of $E_{21}$, the ‘false investigation’ strategy has an expected rate of return of $E_{22}$, and the average expected rate of return is $\bar{E_{2}}$ as follows:

(7) $$E_{21}=xz(I_{t1}-C_{t1})+x(1-z)(I_{t1}-C_{t1})+(1-x)z(I_{t1}-C_{t1})+(1-x)(1-z)(I_{t1}-C_{t1})$$

(8) $$E_{22}=xz(I_{t1}-C_{t2}-P_{t})+x(1-z)(I_{t1}-C_{t2})+(1-x)z(I_{t2}-C_{t2}-P_{t})+(1-x)(1-z)(I_{t2}-C_{t2})$$

(9) $$\bar{E_{2}}=yE_{21}+(1-y)E_{22}$$

Therefore, the replicated dynamic equation of the third party’s ‘true investigation’ strategy is as follows:

(10) $$F(y)=\frac{dy}{dt}=y(E_{21}-\bar{E_{2}})=y(1-y)(C_{t2}-C_{t1}+I_{t1}-I_{t2}-xI_{t1}+xI_{t2}+zP_{t}+xzI_{t1}-xzI_{t2})$$

A partial derivative of the replicated dynamic equation of the third party’s strategy of a ‘true investigation’ can be obtained as follows:

(11) $$\frac{d(F(y))}{dy}=(1-2y)(C_{t2}-C_{t1}+I_{t1}-I_{t2}-xI_{t1}+xI_{t2}+zP_{t}+xzI_{t1}-xzI_{t2})$$

(12) $$\mathrm{We}\;\mathrm{mark}\;J(x)=C_{t2}-C_{t1}+I_{t1}-I_{t2}-xI_{t1}+xI_{t2}+zP_{t}+xzI_{t1}-xzI_{t2}$$

whose derivative is as follows: $\frac{\partial J(x)}{\partial x}=I_{t2}-I_{t1}+zI_{t1}-zI_{t2}=(1-z)(I_{t2}-I_{t1})>0$.

**Figure 3:**
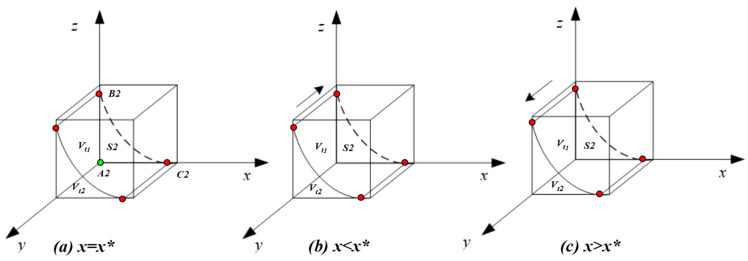
Replicated dynamic phase diagram of the third party.

According to the stability theorem of differential equations, the probability of the third party choosing the ‘true investigation’ strategy must meet the following conditions when it is in a stable state: F(y)=0, d(F(y))/dy<0. Due to ∂J(x)/∂x>0, J(x) will increase with x. It can be obtained through calculation that when $x=\frac{C_{t2}-C_{t1}+I_{t1}-I_{t2}+zP_{t}}{I_{t1}-I_{t2}-zI_{t1}+zI_{t2}}=x*$, J(x)=0, $\frac{d(F(y))}{dy}\equiv 0$, a stable state cannot be achieved; when x>x*, J(x)>0, $\frac{d(F(y))}{dy}{|}_{y=1}<0$, y=1 is an evolutionary stability strategy; and when x<x*, J(x)<0, $\frac{d(F(y))}{dy}{|}_{y=0}<0$, y=0 is an evolutionary stability strategy. Let $J(x)=C_{t2}-C_{t1}+I_{t1}-I_{t2}-xI_{t1}+xI_{t2}+zP_{t}+xzI_{t1}-xzI_{t2}=0$. From this, it is determined that $x=\frac{C_{t2}-C_{t1}+I_{t1}-I_{t2}+zP_{t}}{I_{t1}-I_{t2}-zI_{t1}+zI_{t2}}=x*$. Based on this formula, it can be calculated that $x^{*}$ will pass through point $B_{2}(\frac{C_{t1}-C_{t2}-I_{t1}+I_{t2}}{P_{t}},0)$ and point $C_{2}(0,\frac{C_{t1}-C_{t2}-I_{t1}+I_{t2}}{I_{t2}-I_{t1}})$. Due to $\frac{C_{t1}-C_{t2}-I_{t1}+I_{t2}}{P_{t}}>0$, $\frac{C_{t1}-C_{t2}-I_{t1}+I_{t2}}{I_{t2}-I_{t1}}>0$, an approximate image of the $x^{*}$ function can be obtained (shown in Figure 3). To be specific, subfigure (a) represents the unstable condition when *x* = x*; subfigure (b) shows that *y* = 0 was the ESS when $x<x^{*}$; and subfigure (c) shows that y=1 was the ESS when $x>x^{*}$.

Figure 3 shows the replicator dynamics and evolutionary stability strategies of the third party’s choice of a ‘true investigation’.

As shown in Figure 3, the whole space is divided into region $V_{t1}$ and $V_{t2}$. $V_{t1}$ represents the probability that the third party will select the ‘true investigation’, and $V_{t2}$ represents the probability that the third party will select the ‘false investigation’, as follows: the area formed by $A_{2},B_{2},C_{2}$ is marked as $S_{2}$, which is the planar area of $V_{t2}$($V_{t2}=1*S_{2}$), indicating the $V_{t2}$ will increase as $S_{2}$ increases.

It is calculated that $A_{2}B_{2}=\frac{C_{t1}-C_{t2}-I_{t1}+I_{t2}}{P_{t}}$, $A_{2}C_{2}=\frac{C_{t1}-C_{t2}-I_{t1}+I_{t2}}{I_{t2}-I_{t1}}$, so the larger the values of $A_{2}B_{2}$ and $A_{2}C_{2}$, the larger the values of $S_{2}$ and $V_{t2}$. Due to $V_{t1}=1-V_{t2}$, the smaller the values of $A_{2}B_{2}$ and $A_{2}C_{2}$, the larger the value of $V_{t1}$.

**Inference 3.**  *The probability of a ‘false investigation’ by the third party is related to the costs and incomes of a ‘true investigation’ by the third party, the costs and incomes of a ‘false investigation’, and the government’s fines imposed on the third party. Specifically, the probability of a ‘false investigation’ by the third party is positively related to the cost of a ‘true investigation‘ and the incomes of a ‘false investigation’, and negatively related to the cost of a ‘false investigation’, the incomes of a true investigation, and the government’s fines imposed on the third party. In other words, the probability of the third party’s ‘true investigation’ is negatively related to the cost of a ‘true investigation’ and the incomes of the ‘false investigation’, while it is positively related to the cost of a ‘false investigation’, the incomes of a ‘true investigation’, and the government’s fines imposed on the third party.*

**Proof.**  The first order partial derivative of each element for $A_{2}B_{2}$ is calculated as follows:

$$\partial A_{2}B_{2}/\partial C_{t1}>0,\partial A_{2}B_{2}/\partial C_{t2}<0,\;\partial A_{2}B_{2}/\partial I_{t1}<0,\;\partial A_{2}B_{2}/\partial I_{t2}>0,\;\partial A_{2}B_{2}/\partial P_{t}<0$$

Based on the positive relationship between $A_{2}B_{2}$ and $V_{t2}$, it can be concluded that

$$\partial V_{t2}/\partial C_{t1}>0,\partial V_{t2}/\partial C_{t2}<0,\;\partial V_{t2}/\partial I_{t1}<0,\;\partial V_{t2}/\partial I_{t2}>0,\;\partial V_{t2}/\partial P_{t}<0$$

Due to $V_{t1}=1-V_{t2}$, it can be inferred that

$$\partial V_{t1}/\partial C_{t1}<0,\;\partial V_{t1}/\partial C_{t2}>0,\;\partial V_{t1}/\partial I_{t1}>0,\;\partial V_{t1}/\partial I_{t2}<0,\;\partial V_{t1}/\partial P_{t}>0$$

From Inference 3, it can be seen that reducing the cost of the third party’s ‘true investigation’ or increasing the cost of the third party’s ‘false investigation’ can drive the third party to choose the strategy of the ‘true investigation’. In the same way, increasing the incomes of a third party’s ‘true investigation’ or reducing the incomes of its ‘false investigation’ can encourage the third party to choose a ‘true investigation’ strategy. In addition, local governments’ increasing of incentives to the third parties can drive them to choose the strategy of a ‘true investigation’. □

**Inference 4.**  *The probability of a ‘true investigation’ by the third party will increase with the increase of the probability of the ‘compliant operation’ of the hospital or the probability of ‘strict supervision’ by the local government.*

**Proof.**  It can be obtained from the strategic stability of third party that

When $x<\frac{C_{t2}-C_{t1}+I_{t1}-I_{t2}+zP_{t}}{\left(I_{t1}-I_{t2})(1-z\right)}$ or $z<\frac{C_{t1}-C_{t2}-I_{t1}+I_{t2}+xI_{t1}-xI_{t2}}{P_{t}+xI_{t1}-xI_{t2}}$, J(x)<0, $\frac{d(F(y))}{dy}{|}_{y=0}<0$, y=0 will be an evolutionary stabilization strategy (the ESS point). When $x>\frac{C_{t2}-C_{t1}+I_{t1}-I_{t2}+zP_{t}}{\left(I_{t1}-I_{t2})(1-z\right)}$ or $z>\frac{C_{t1}-C_{t2}-I_{t1}+I_{t2}+xI_{t1}-xI_{t2}}{P_{t}+xI_{t1}-xI_{t2}}=z*$, J(x)>0, $\frac{d(F(y))}{dy}{|}_{y=1}<0$, y=1 will be an evolutionary stabilization strategy (the ESS point). In other words, the larger the values of x,z,the larger the value of y.

It can be concluded from inference 4 that when the probability of compliant operation of hospital is low, the third party will be prompted to adopt the stable strategy of a false investigation. When the probability of strict supervision by the local government increases, it will also urge the third party to adopt the stable strategy of a true investigation. Because when the probability of compliant operation of hospital is low, the probability of hospital bribing the third party is higher, so the third party can obtain additional rent-seeking benefits while the cost of strict supervision by the local government is very high. If the local government chooses a non-supervision strategy, the third party will not be fined, so the third party will also choose a stable strategy of a false investigation. In addition, when the local government takes a strict supervision strategy, the third party will be fined if conducting a false investigation, so the third party will also tend to choose a stable strategy of true investigation. □

### 4.3. Government Evolution Stability Strategy

Assuming that the local government chooses a ‘strict supervision’ strategy with an expected rate of return of $E_{31}$, the ‘non-supervision’ strategy has an expected rate of return of $E_{32}$, and the average expected rate of return is $\bar{E_{3}}$ as follows:

(13) $$E_{31}=xy(I_{g1}-C_{g}-A_{h}+A_{g})+x(1-y)(I_{g1}-C_{g}-A_{h}+P_{t}+A_{g})+(1-x)y(I_{g2}-C_{g}+P_{h}+A_{g})$$

(14) $$E_{32}=xy(I_{g1})+x(1-y)(I_{g1})+(1-x)y(I_{g2}+P_{h})+(1-x)(1-y)(I_{g2}-P_{g})$$

(15) $$\bar{E_{3}}=zE_{31}+(1-z)E_{32}$$

Therefore, the replicated dynamic equation of the local government’s strict ‘supervision’ strategy is as follows:

(16) $$F(z)=\frac{dz}{dt}=z(E_{31}-\bar{E_{3}})=z(z-1)(C_{g}-A_{g}-P_{g}-P_{h}-P_{t}+xA_{h}+xP_{g}+xP_{h}+yP_{g}+yP_{h}+yP_{t}-xyP_{g}-xyP_{h})$$

A partial derivative of the replicated dynamic equation of the government’s choice of strict ‘supervision’ strategy can be obtained as follows:

(17) $$\frac{dF(z)}{dz}=(2z-1)(C_{g}-A_{g}-P_{g}-P_{h}-P_{t}+xA_{h}+xP_{g}+xP_{h}+yP_{g}+yP_{h}+yP_{t}-xyP_{g}-xyP_{h})$$

(18) $$\mathrm{We}\;\mathrm{mark}\;H(y)=(C_{g}-A_{g}-P_{g}-P_{h}-P_{t}+xA_{h}+xP_{g}+xP_{h}+yP_{g}+yP_{h}+yP_{t}-xyP_{g}-xyP_{h})$$

whose derivative is as follows: $\partial H(y)/\partial y=P_{g}+P_{h}+P_{t}-x(P_{g}+P_{h})=(P_{g}+P_{h})(1-x)+P_{t}>0$.

**Figure 4:**
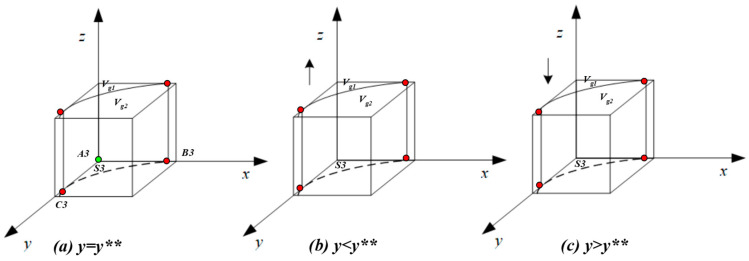
Replicated dynamic phase diagram of the local government.

According to the stability theorem of differential equations, the probability of local government choosing a strict supervision strategy must meet the following conditions when it is in a stable state:

F(z)=0 and dF(z)/dz<0. Due to ∂H(y)/∂y>0, H(y) will increase with y. It can be obtained through calculation that when $y=\frac{A_{g}-C_{g}+P_{g}+P_{h}+P_{t}-xA_{h}-xP_{g}-xP_{h}}{P_{g}+P_{h}+P_{t}-x(P_{g}+P_{h})}=y**$, H(y)=0, $\frac{d\left(F(z)\right)}{dz}\equiv 0$, a stable state cannot be achieved; when y<y**, H(y)<0, $d(F(z))/dz{|}_{z=1}<0$, z=1 is an evolutionary stability strategy; and when y>y**, H(y)>0, $d(F(z))/dz{|}_{z=0}<0$, z=0 is an evolutionary stability strategy. Let $H(y)=(C_{g}-A_{g}-P_{g}-P_{h}-P_{t}+xA_{h}+xP_{g}+xP_{h}+yP_{g}+yP_{h}+yP_{t}-xyP_{g}-xyP_{h})=0$. From this, it is determined that $y=\frac{A_{g}-C_{g}+P_{g}+P_{h}+P_{t}-xA_{h}-xP_{g}-xP_{h}}{P_{g}+P_{h}+P_{t}-x(P_{g}+P_{h})}=y**$. Based on this formula, it can be calculated that $y^{**}$ will pass through point $C_{3}(0,\frac{A_{g}-C_{g}+P_{g}+P_{h}+P_{t}}{P_{g}+P_{h}+P_{t}})$ and point $B_{3}(\frac{A_{g}-C_{g}+P_{g}+P_{h}+P_{t}}{A_{h}+P_{g}+P_{h}},0)$. Since $\frac{A_{g}-C_{g}+P_{g}+P_{h}+P_{t}}{P_{g}+P_{h}+P_{t}}$ and $\frac{A_{g}-C_{g}+P_{g}+P_{h}+P_{t}}{A_{h}+P_{g}+P_{h}}$ have same symbol, an approximate image of the $y^{**}$ function can be obtained (shown in Figure 4). To be specific, subfigure (a) represents the unstable condition when $y=y^{**}$; subfigure (b) shows that z = 1 was the ESS when $y<y^{**}$; and subfigure (c) shows that z = 0 was the ESS when $y>y^{**}$.

Figure 4 shows the replicator dynamics and evolutionary stability strategies of the government’s choice of ‘strict supervision’.

As shown in Figure 4, the whole space is divided into region $V_{g1}$ and region $V_{g2}$. $V_{g1}$ represents the probability that the government will select ‘strict supervision’, and $V_{g2}$ represents the probability that the government will select ‘non-supervision’, as follows:

The area formed by $A_{3},B_{3},C_{3}$ is marked as $S_{3}$, which is the planar area of $V_{g1}$($V_{g1}=1*S_{3}$), indicating the $V_{g1}$ will increase as $S_{3}$ increases. It is calculated that $A_{3}B_{3}=\frac{A_{g}-C_{g}+P_{g}+P_{h}+P_{t}}{A_{h}+P_{g}+P_{h}}$, $A_{3}C_{3}=\frac{A_{g}-C_{g}+P_{g}+P_{h}+P_{t}}{P_{g}+P_{h}+P_{t}}$, so the larger the values of $A_{3}B_{3}$ and $A_{3}C_{3}$, the larger the values of $S_{3}$ and $V_{g1}$.

**Inference 5.**  *The probability of ‘strict supervision’ by local government is related to the reward of the superior government, the cost of strict supervision, the punishment of the superior government, the fines imposed on the hospital, the fines imposed on the third party, and the reward given to hospital. Specifically, the probability of ‘strict supervision’ by local government is positively related to the reward of the superior government, the punishment of the superior government, the fines imposed on the hospital, and the fines imposed on the third party, while it is negatively related to the reward given to the hospital and the cost of ‘strict supervision’ by the government.*

**Proof.**  The first order partial derivative of each element for $A_{3}B_{3}$ is calculated as follows:

$$\partial A_{3}B_{3}/\partial A_{g}>0,\;\partial A_{3}B_{3}/\partial P_{t}>0,\;\partial A_{3}B_{3}/\partial C_{g}<0,\;\partial A_{3}B_{3}/\partial A_{h}<0,\;\partial A_{3}B_{3}/\partial P_{g}>0,\;\partial A_{3}B_{3}/P_{h}>0$$

Based on the positive relationship between $A_{3}B_{3}$ and $V_{g1}$, it can be concluded that

$$\partial V_{g1}/\partial A_{g}>0,\;\partial V_{g1}/\partial P_{t}>0,\;\partial V_{g1}/\partial C_{g}<0,\;\partial V_{g1}/\partial A_{h}<0,\;\partial V_{g1}/\partial P_{g}>0,\;\partial V_{g1}/\partial P_{h}>0$$

From inference 5, it can be concluded that the superior government can encourage the local government to adopt a strict supervision strategy by increasing the rewards and punishments to the local government. In addition, increasing the fines imposed by the local government on the hospital and the third party can also increase the probability that the local government chooses to conduct strict supervision. On the contrary, raising the cost of strict supervision by the local government and the reward on the hospital will reduce the probability of strict supervision by the local government. □

**Inference 6.**  *The probability of the ‘strict supervision’ by the local government will increase with the increase of the probability of the ‘illegal operation’ of the hospital and the probability of a ‘false investigation’ by the third party.*

**Proof.**  It can be obtained from the strategic stability of local government that

When $y<\frac{A_{g}-C_{g}+P_{g}+P_{h}+P_{t}-xA_{h}-xP_{g}-xP_{h}}{P_{g}+P_{h}+P_{t}-x(P_{g}+P_{h})}$ or $x<\frac{A_{g}-C_{g}+P_{g}+P_{h}+P_{t}-yP_{g}-yP_{h}-yP_{t}}{A_{h}+P_{g}+P_{h}-yP_{g}-yP_{h}}$, H(y)<0, $d(F(z))/dz{|}_{z=1}<0$, z=1 will be an evolutionary stabilization strategy (the ESS point). When $y>\frac{A_{g}-C_{g}+P_{g}+P_{h}+P_{t}-xA_{h}-xP_{g}-xP_{h}}{P_{g}+P_{h}+P_{t}-x(P_{g}+P_{h})}$ or $x>\frac{A_{g}-C_{g}+P_{g}+P_{h}+P_{t}-yP_{g}-yP_{h}-yP_{t}}{A_{h}+P_{g}+P_{h}-yP_{g}-yP_{h}}$, H(y)>0, $d(F(z))/dz{|}_{z=0}<0$, z=0 will be an evolutionary stabilization strategy (the ESS point). In other words, the larger the values of x,y, the smaller the value of y.

From Inference 6, it can be concluded that the illegal operation of the hospital and the false investigation by the third party can drive the local government to adopt a strict supervision strategy. The compliance operation of the hospital and the true investigation of the third party will result in non-supervision from the local government. According to the analysis, if the hospital operates in violation of regulations or the third party conducts a false investigation, the local government, using strict supervision, will impose fines on the hospital or the third party so as to improve the benefits to the local government. If the hospital operates in compliance with the regulations, the local government can obtain higher social benefits. Additionally, the true investigation of the third party can help the local government find the problems in the hospital in time, so the local government will tend to take a non-supervision strategy under the circumstance of a high supervision cost. □

### 4.4. Stability Analysis of Equilibrium Point of Tripartite Evolutionary Game System

According to the method proposed by Friedman, the evolutionary stability strategy (ESS) of a differential equation system can be obtained from the local stability analysis of the Jacobian matrix [47]. Let F(x)=0,F(y)=0,F(z)=0. From this, we can obtain a total of 15 equilibrium points, of which there are eight pure strategy equilibrium points. Then, the next step is to substitute each equilibrium point into the matrix to obtain the corresponding Jacobian matrix of each equilibrium point. The eigenvalues of each matrix are shown in Table 3. It is indicated that the asymptotically stable solution of the multi-agent evolutionary game system must be a strict Nash equilibrium and a pure strategy Nash equilibrium [48], thus $A_{9}\sim A_{15}(x*,y*,z*)$ will not be considered.

$$J=\left[\begin{array}{l} J_{1}\;J_{2}\;J_{3}\\ J_{4}\;J_{5}\;J_{6}\\ J_{7}\;J_{8}\;J_{9} \end{array}\right]=\left[\begin{array}{l} \partial F(x)/\partial x\partial F(x)/\partial y\partial F(x)/\partial z\\ \partial F(y)/\partial x\partial F(y)/\partial y\;\partial F(y)/\partial z\\ \partial F(z)/\partial x\partial F(z)/\partial y\partial F(z)/\partial z \end{array}\right]$$

$$=\left[\begin{array}{lll} \begin{array}{l} (1-2x)\;(C_{h2}-C_{h1}+I_{h1}-I_{h2}\\ +zA_{h}+yP_{h}+zP_{h}-yzP_{h}), \end{array} & x(x-1)(z-1)P_{h},\; & x(1-x)(A_{h}+P_{h}-yP_{h});\\ -y(y-1)(z-1)(I_{t1}-I_{t2}), & \begin{array}{l} -(1-2y)\;(C_{t2}-C_{t1}+I_{t1}-I_{t2}\\ -xI_{t1}+xI_{t2}+zP_{t}+xzI_{t1}-xzI_{t2}), \end{array} & -y(y-1)(P_{t}+xI_{t1}-xI_{t2});\\ z(z-1)(A_{h}+P_{g}+P_{h}-yP_{g}-yP_{h}), & z(z-1)(P_{g}+P_{h}+P_{t}-xP_{g}-xP_{h}), & \begin{array}{l} (2z-1)(C_{g}-A_{g}-P_{g}-P_{h}-P_{t}+xA_{h}+xP_{g}\\ +xP_{h}+yP_{g}+yP_{h}+yP_{t}-xyP_{g}-xyP_{h});\; \end{array} \end{array}\right]$$

By the first Lyapunov method [49], the equilibrium point can be determined by analyzing the eigenvalues of the Jacobian matrix. First, when all three eigenvalues of the Jacobian matrix have a negative real part, the corresponding equilibrium point is the ESS. Second, when all the three eigenvalues have a positive real part, the equilibrium is an unstable point. Finally, when one or two eigen values are positive, the equilibrium point is a saddle point. The Jacobian matrix of the dynamic tripartite game can be obtained as follows:

Based on the analysis of each eigenvalue symbol, it can be confirmed that the points $E_{2}$, $E_{3}$, $E_{5}$, $E_{7}$, and $E_{8}$ are definitely not equilibrium points. The reasons are follows:

For the eigenvalue of $E_{2}$, because $C_{h1}>C_{h2}$, $I_{h2}>I_{h1}$, indicating that $\lambda_{3}=C_{h1}-C_{h2}-I_{h1}+I_{h2}>0$, $E_{2}$ must not be the ESS. For the eigenvalue of $E_{3}$, because $C_{t1}>C_{t2}$, $I_{t2}>I_{t1}$, indicating that $\lambda_{2}=C_{t1}-C_{t2}-I_{t1}+I_{t2}>0$, *E*_3_ must not be the ESS. For the eigenvalue of $E_{5}$, because $\lambda_{1}=C_{t1}-C_{t2}>0$, $E_{5}$ must not be the ESS.

For the eigenvalue of $E_{7}$, because $\lambda_{1}=C_{g}-A_{g}>0$, $E_{7}$ must not be the ESS. For the eigenvalue of $E_{8}$, because $C_{g}>A_{g}$, $A_{h}>0$, indicating that $\lambda_{1}=A_{h}-A_{g}+C_{g}>0$, $E_{8}$ must not be the ESS.

Therefore, only point $E_{1}$, $E_{4}$, and $E_{6}$ can be the ESS point under certain conditions. For point $E_{1}$, when $C_{g}>A_{g}+P_{g}+P_{h}+P_{t}\;$, it will be the ESS; for point $E_{4}$, when $P_{t}<C_{t1}-C_{t2}+I_{t2}-I_{t1}$ and $A_{h}+P_{h}<C_{h1}-C_{h2}+I_{h2}-I_{h1}$, it will be the ESS; and for point $E_{6}$, when $A_{h}+C_{g}<A_{g}+P_{t}$, it will be the ESS.

## 5. Numerical Simulation Analysis

Based on the above model and the theoretical analysis, once certain conditions are met, the system will reach a stable state. In order to intuitively express the evolution trend of each equilibrium point, a numerical simulation is used.

### 5.1. The Numerical Simulation Results under Scenario 1 (0,0,0)

The equilibrium conditions for the game behavior of all subjects are.

$$\lambda 1=C_{h2}-C_{h1}+I_{h1}-I_{h2}<0,\;\lambda 2=C_{t2}-C_{t1}+I_{t1}-I_{t2}<0,\;\lambda 3=A_{g}-C_{g}+P_{g}+P_{h}+P_{t}<0$$

**Figure 5:**
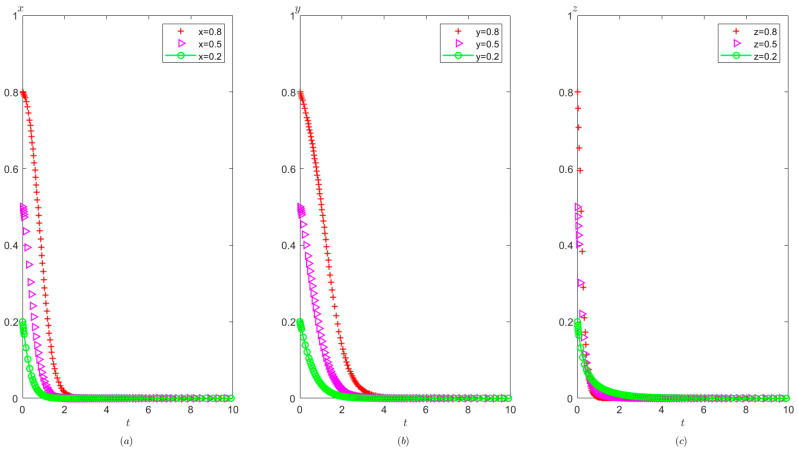
Evolutionary process of the behavior of the hospital, the third party, and the local government under scenario 1: (**a**) the hospital, (**b**) the third party, and (**c**) the local government.

Suppose $C_{h1}=8,C_{h2}=5,C_{t1}=2,C_{t2}=1,C_{g}=9,I_{h1}=9,I_{h2}=10,I_{t1}=3,I_{t2}=4,I_{g1}=8,I_{g2}=5,A_{h}=2,$ $P_{h}=2,P_{t}=1,A_{g}=2,P_{g}=3$. The initial probability value states of the hospital, the third party, and the local government are 0.8, 0.5, and 0.2, respectively. The evolution time is set to T = [0, 10]. The numerical simulation results are shown in Figure 5a–c, indicating that whatever the initial states of the hospital, the third party, and the local government, they will eventually evolve to (0,0,0). The strategy evolution process of each agent is shown in Figure 5.

It can be observed from Figure 5 that regardless of the probability of the initial strategy of the hospital, the third party, and the local government, their strategy choice will eventually be stabilized in the illegal operation, false investigation, and non-supervision, respectively. That is, as long as the income from the hospital’s illegal operation is greater than the income from the compliant operation, the hospital will tend to operate in violation of regulations. Similarly, as long as the income of the third party’s false investigation is more than the income from the true investigation, the third party will tend to conduct false investigations. In addition, when the cost of strict supervision by the local government is very high, that is, s higher than the sum of the rewards and fines from the superior government as well as the fines imposed on the hospital and the third party, the local government will tend to take a non-supervision strategy.

**Figure 6:**
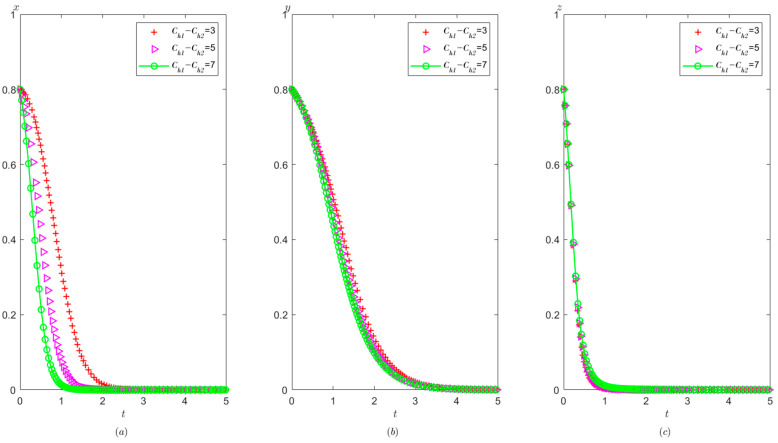
The effects of $C_{h1}-C_{h2}$ on evolutionary process of the behavior of hospital, the third party, and local government under scenario 1: (**a**) the hospital, (**b**) the third party, and (**c**) the local government.

It can be seen from Figure 6 that in the process of system evolution to a stable point, the larger the cost difference between compliant operation and illegal operation of the hospital $C_{h1}-C_{h2}$, the faster the stabilization of the hospital’s illegal operations. However, $C_{h1}-C_{h2}$ has no significant impact on the stability strategies of the third party and the local government.

**Figure 7:**
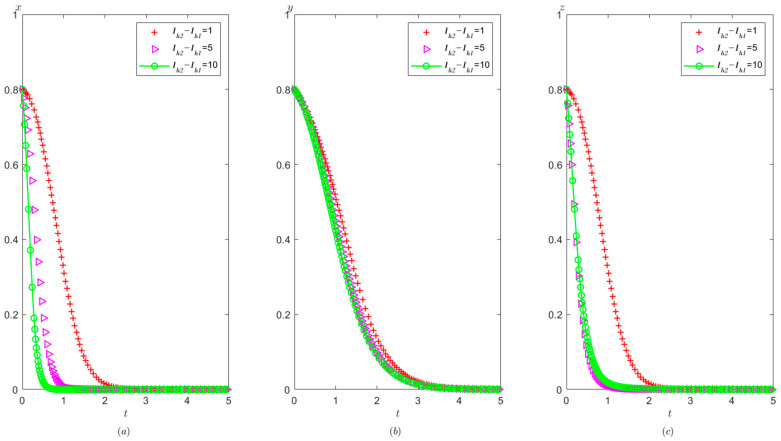
The effects of $I_{h2}-I_{h1}$ on evolutionary process of the behavior of hospital, the third party, and local government under scenario 1: (**a**) the hospital, (**b**) the third party, and (**c**) the local government.

It can be seen from Figure 7 that in the process of strategy evolution, with the increase of $I_{h2}-I_{h1}$, the hospital tends to operate illegal business faster. The speed at which the local government tends to adopt non-supervision has also accelerated and the speed at which the third party tends to conduct false investigation has not changed significantly.

**Figure 8:**
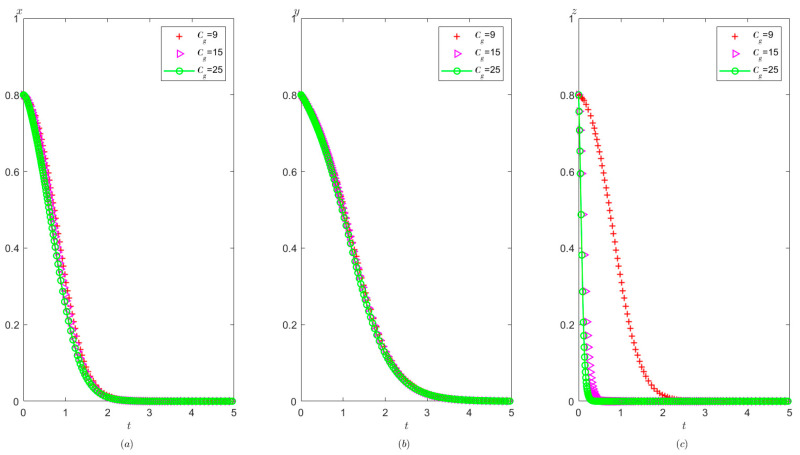
The effects of $C_{g}$ on evolutionary process of the behavior of hospital, the third party, and the local government under scenario 1: (**a**) the hospital, (**b**) the third party, and (**c**) the local government.

It can be seen from Figure 8 that in the process of strategy evolution, with the increase in $C_{g}$, the local government evolves to adopt a non-supervision strategy more quickly, but the cost of strict supervision by the local government has no obvious impact on the evolution speed of the hospital and the third party.

### 5.2. The Numerical Simulation Results under Scenario 2 (0,0,1)

The equilibrium conditions for the game behavior of both subjects are

$$\lambda 1=C_{g}-A_{g}-P_{g}-P_{h}-P_{t}<0,\lambda 2=C_{t2}-C_{t1}+I_{t1}-I_{t2}+P_{t}<0,\lambda 3=A_{h}-C_{h1}+C_{h2}+I_{h1}-I_{h2}+P_{h}<0$$

**Figure 9:**
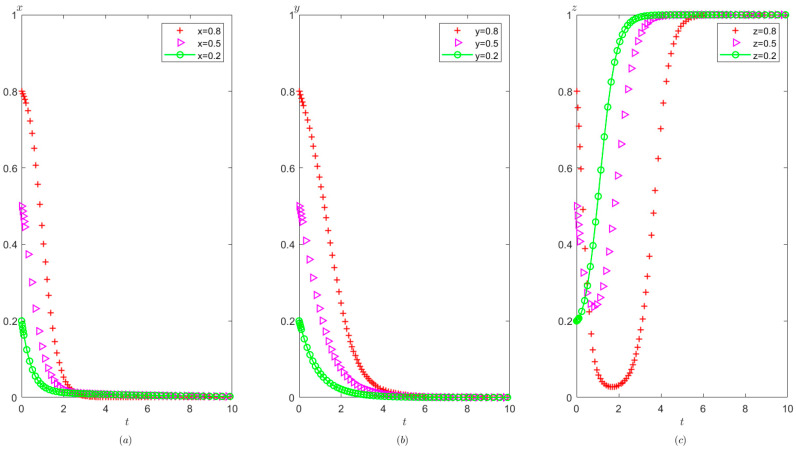
Evolutionary process of the behavior of hospital, the third party, and the local government under scenario 2: (**a**) the hospital, (**b**) the third party, and (**c**) the local government.

Suppose $C_{h1}=7,C_{h2}=5,C_{t1}=2,C_{t2}=1,C_{g}=5.5,I_{h1}=8,I_{h2}=9,I_{t1}=2.5,I_{t2}=3,I_{g1}=8,I_{g2}=5,A_{h}=2,$ $P_{h}=0.8,P_{t}=0.5,A_{g}=2,P_{g}=5$. The initial probability value states of the hospital, the third party, and the local government are all 0.8, 0.5, and 0.2, respectively. The evolution time is set to T = [0, 10]. The numerical simulation results are shown in Figure 9a–c, indicating that whatever the initial states of the hospital, the third party and local governments, they will eventually evolve to (0,0,1).

It can be obtained from Figure 9 that regardless of the probability of the initial strategy of the hospital, the third party, and the local government, their strategy choice will eventually be stabilized in the illegal operation, false investigation, and strict supervision. That is, when the sum of the local government’s rewards and punishments to the hospital is less than the difference between the profits of the illegal operation and the profits of the compliant operation, the hospital will tend to operate in violation of regulations. When the fine on the third party is less than the benefit difference between the false investigation and the true investigation, the third party will tend to make a false investigation. When the cost of strict supervision by the local government is less than the sum of the rewards and fines from the superior government and fines imposed on the hospital and the third party, the local government will tend to use strict supervision.

Compared with Figure 5 and Figure 9, when the cost of strict supervision by the local government changes from $C_{g}>A_{g}+P_{g}+P_{h}+P_{t}$ to $C_{g}<A_{g}+P_{g}+P_{h}+P_{t}$, the local government’s strategy choice will change from non-supervision to strict supervision.

**Figure 10:**
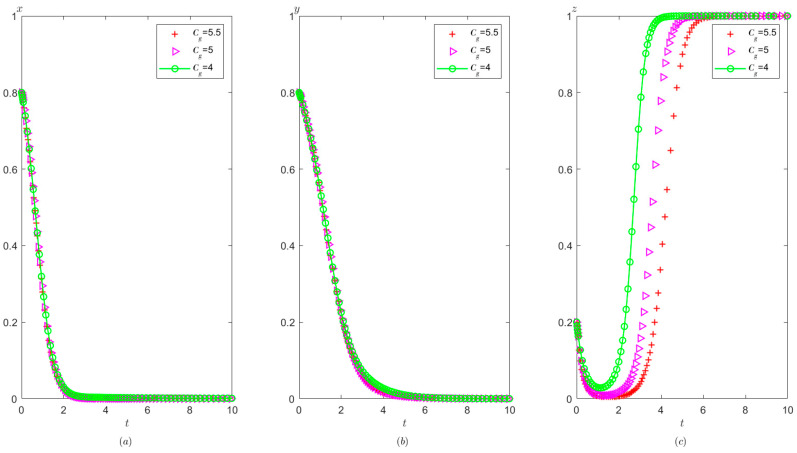
The effects of $C_{g}$ on evolutionary process of the behavior of hospital, the third party, and the local government under scenario 2: (**a**) the hospital, (**b**) the third party, and (**c**) the local government.

As shown in Figure 10, the equilibrium strategies of the hospital, the third party, and the local governments are (0,0,1). Under these circumstances, with the increase in supervision cost for the government, the speed of the government’s stabilization to strict supervision is slower. The speed at which the hospital tends to operate in violation of regulations and the speed at which the third party tends to conduct false investigations has not changed significantly.

**Figure 11:**
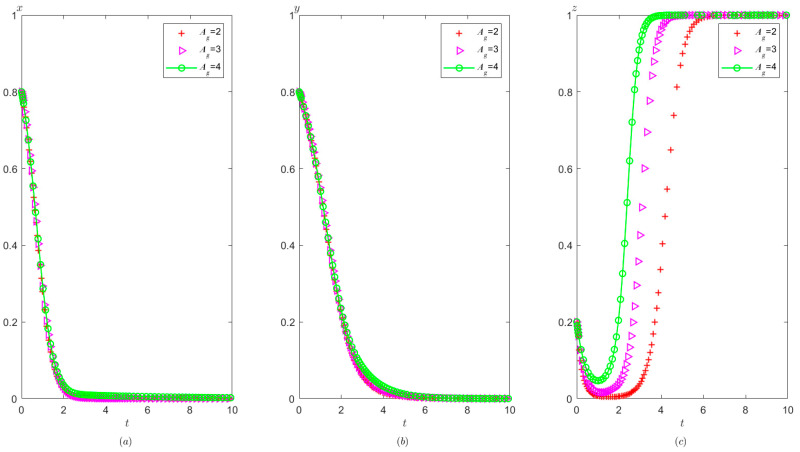
The effects of $A_{g}$ on evolutionary process of the behavior of hospital, the third party, and local government under scenario 2: (**a**) the hospital, (**b**) the third party, and (**c**) the local government.

As shown in Figure 11, with the improvement of the local government’s reward from the superior government $A_{g}$, the local government tends to take strict supervision faster, but the speed at which the hospital tends to conduct illegal operations and the speed at which the third party tends to conduct false investigations have not changed significantly.

**Figure 12:**
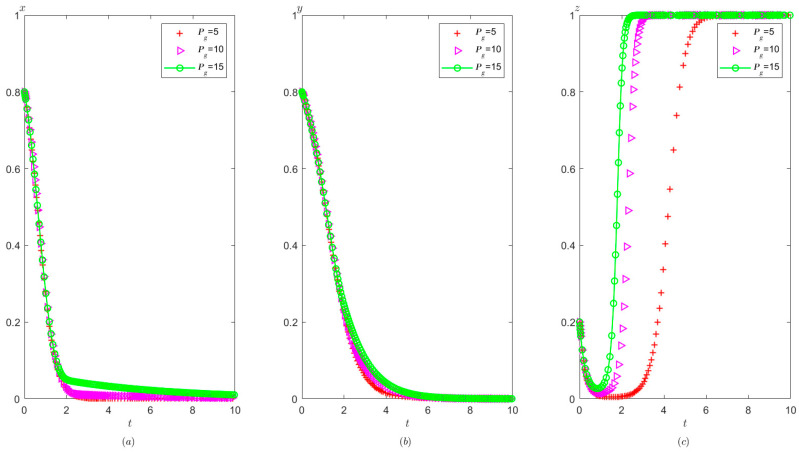
The effects of $P_{g}$ on evolutionary process of the behavior of hospital, the third party, and the local government under scenario 2: (**a**) the hospital, (**b**) the third party, and (**c**) the local government.

As shown in Figure 12, the greater the penalty of the superior government on the local government $P_{g}$, the faster the tendency of the local government toward adopting a supervision strategy, while the speed at which the hospital tends to operate in violation of regulations and the speed at which third party tends to conduct false investigation strategy has no significant change.

### 5.3. The Numerical Simulation Results under Scenario 3 (1,0,1)

The equilibrium conditions for the game behavior of both subjects are

$$\lambda 1=A_{h}-A_{g}+C_{g}-P_{t}<0,\lambda 2=C_{t2}-C_{t1}+I_{t1}-I_{t2}+P_{t}<0,\lambda 3=C_{h1}-A_{h}-C_{h2}-I_{h1}+I_{h2}-P_{h}<0$$

**Figure 13:**
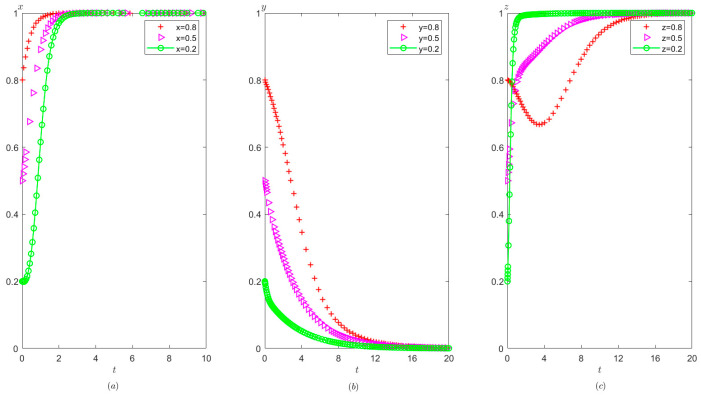
Evolutionary process of the behavior of hospital, the third party, and the local government under scenario 3: (**a**) the hospital, (**b**) the third party, and (**c**) the local government.

Suppose $C_{h1}=6,C_{h2}=4.5,C_{t1}=2,C_{t2}=1,C_{g}=5,I_{h1}=6.5,I_{h2}=7,I_{t1}=2.5,I_{t2}=3,I_{g1}=5.5,I_{g2}=4,A_{h}=0.6,$ $P_{h}=4.5,P_{t}=1.2,A_{g}=4.9,P_{g}=3$. The initial probability value states of the hospital, the third party, and the local government are all 0.8, 0.5, and 0.2, respectively. The evolution time is set to Ta = [0, 10], Tb = [0, 20], and Tc = [0, 20], respectively. The numerical simulation results are shown in Figure 13a–c, indicating that whatever the initial states of hospital, the third party, and the local government, they will eventually evolve to (1,0,1).

It can be seen from Figure 13 that regardless of the probability of the initial strategy of the hospital, the third party, and the local government, their strategy choices will eventually stabilize in compliance operation, false investigation, and strict supervision, respectively. That is, when the sum of the local government’s reward and punishment to the hospital is greater than the difference between the benefit from illegal operation and compliant operation, the hospital will tend to operate in compliance. When the fine on the third party is less than the benefit difference between the false investigation and the true investigation, the third party will tend to make a false investigation. When the sum of the cost of strict supervision by the local government and the reward given to the hospital is less than the sum of the reward given by the superior government to the local government and the fine imposed on the third party, the local government will tend to conduct strict supervision.

From Figure 9 and Figure 13, it can be concluded that when $A_{h}+P_{h}<C_{h1}-C_{h2}-I_{h1}+I_{h2}$ changes to $A_{h}+P_{h}>C_{h1}-C_{h2}-I_{h1}+I_{h2}$, the hospital’s strategy will change from illegal operation to compliant operation.

**Figure 14:**
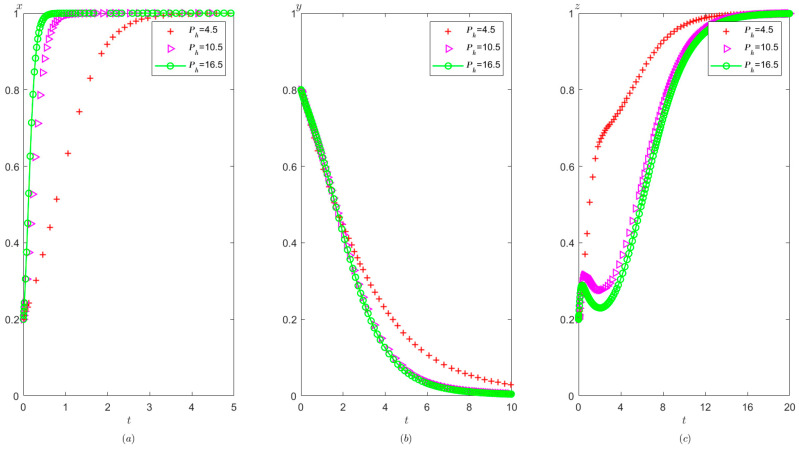
The effects of $P_{h}$ on evolutionary process of the behavior of hospital, the third party, and local government under scenario 3: (**a**) the hospital, (**b**) the third party, and (**c**) the local government.

It is shown in Figure 14 that with the improvement of the local government’s penalty on the hospital, the faster the hospital tends to operate in compliance, and the faster the third party tends to conduct false investigations, but the speed at which the local government stabilizes at strict supervision becomes slower. According to the analysis, when the local government increases the penalties for illegal operation of the hospital, the probability of compliant operation of the hospital is significantly increased. In this context, the third party tends to make false investigations in order to save its own investigation costs. Similarly, a high fine will urge the hospital to operate in compliance with the regulations. At the same time, the local government will relax its vigilance, thus resulting in a slower speed of stabilization at strict supervision.

**Figure 15:**
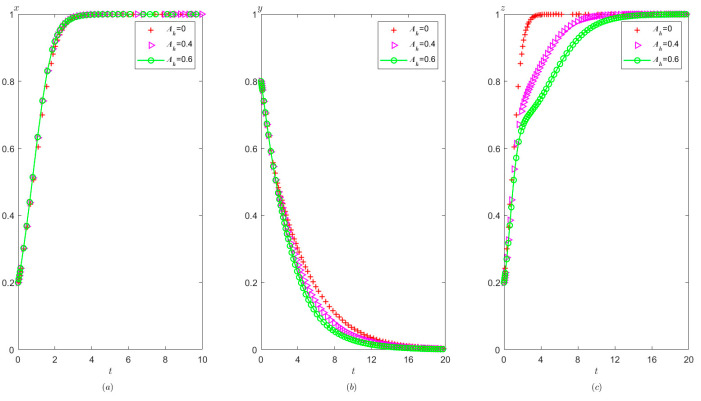
The effects of $A_{h}$ on evolutionary process of the behavior of hospital, the third party, and local government under scenario 3: (**a**) the hospital, (**b**) the third party, and (**c**) the local government.

It is indicated from Figure 15 that on the basis of meeting condition $C:A_{h}+C_{g}<A_{g}+P_{t}$, the local government’s reward for the hospital has no significant impact on the probability of the hospital tending to operate in compliance, but it slightly increases the speed at which the third party evolve to conduct false investigation and reduces the speed at which local governments evolve to adopt strict supervision.

## 6. Conclusions

Based on the common experience of healthcare fraud, this paper constructs a tripartite evolutionary game model among the hospital, the third party, and the local government, systematically analyzing the stability of participants’ strategy selection and the stability of the equilibrium strategy combination of the game and investigating the influencing factors of equilibrium strategy selection using a numerical simulation. The following main conclusions are obtained. First, in the process of compliant operation by the hospital, the local government’s fines on the hospital are the key factor. The punishment mechanism exerted by the local government can drive the hospital to increase the probability of legal operation. The higher the fines, the faster the hospital tends to operate in compliance. In this situation, the local government also tends to be strict in supervision, and the promotion of incentives for the hospital has less impact on the compliant operation of the hospital. Because excessive incentives for the hospital will increase the economic pressure on the local government, by inhibiting the probability of strict supervision from the local government, the local government will offer fewer incentives to the hospital with the compliant operation. In this context, the punishment mechanism plays a major role. Secondly, the cost and benefit of the hospital are also the key factors affecting their strategic choices. The high cost of compliant operation will inhibit the probability of compliant operation of the hospital, and the low benefit of compliant operation will also reduce the probability of compliance operation. Therefore, it is necessary to coordinate all parties and innovate the management mode of hospital to reduce their operating costs.

In addition, the cost and benefit of strict supervision by the local government are critical factors affecting the local government’s strategic choices. The high supervision cost of the local government will restrain the probability of their strict supervision strategy, and the rewards from the superior government will encourage the local government to strictly supervise the behavior of hospital. Accordingly, the superior government needs to increase the incentives to the local government and encourage the local government to improve the level of supervision so as to reduce the cost of strict supervision. Therefore, from a public health perspective, establishing a sound regulatory mechanism is necessary for the management of healthcare. This includes the construction of regulatory departments, regulations and procedures, and other aspects to ensure the effectiveness and scientific nature of regulatory work. Specifically, the provision and management of healthcare require a sound regulatory mechanism, strengthened supervision and auditing of hospital behavior, improved independence and professionalism of the third-party regulatory institutions, enhanced local government oversight, and increased public participation. Together these efforts will ensure the safe and effective use of healthcare and provide guarantees for public health and well-being.

## 7. Implications

The analysis of the evolutionary game between hospitals, third parties, and the government concerning healthcare fraud carries both theoretical and practical significance. Theoretical implications arise from its ability to deepen our understanding of healthcare fraud by revealing the interactions and strategic choices among these entities. It also aids in exploring evolutionarily stable strategies, shedding light on advantageous long-term approaches, and informing effective countermeasures and policies.

From a practical standpoint, this analysis offers valuable insights for policy formulation and governance. It provides recommendations to combat healthcare fraud, enhance integrity, and improving efficiency in healthcare systems. By identifying the key factors affecting the behaviors of different individuals, it assists in developing targeted prevention measures and regulatory mechanisms. Furthermore, the study of evolutionary game dynamics uncovers mechanisms that promote cooperation and coordination among hospitals, third parties, and the government, fostering a collaborative approach to collectively address healthcare fraud. Lastly, understanding the economic impact of healthcare fraud facilitates resource optimization and informed decision-making within healthcare systems.

In summary, the analysis of the evolutionary game between hospitals, third-parties, and the government regarding healthcare fraud holds significant theoretical implications for understanding fraud dynamics and ethical behavior. Moreover, it carries practical implications for policy-making, risk prevention, cooperation mechanisms, and resource optimization within healthcare systems.

## 8. Limitations

There are a few limitations to this paper. Firstly, the evolutionary game method possesses certain drawbacks. It tends to simplify model parameters and overlook the heterogeneity among individuals involved in the game. Moreover, this paper lacks an analysis of a reward mechanism for the third party. Against this background, the third party will not choose a stable strategy of true investigation. If the fines on the third party are increased, the strategies of the third party and the local government cannot reach to a stable state. Therefore, additional research can further consider the incentive mechanism for the third party. Finally, as patients are also one of the main participants in healthcare fraud, patient’s behavior choice in the game relationship must be determined. Due to the complexity of the evolutionary game involving four parties, this paper does not study the patient’s behavior choice in the game relationship. Future research should take patients into account and study the behavior strategy choice of all of the four parties.

## Figures and Tables

**Table 1:** Variables and description of evolutionary games.

Variables	Connotations
$C_{h1}$	Cost of compliant operation of hospital
$C_{h2}$	Cost of illegal operation of hospital
$C_{t1}$	Cost of true investigation by third party
$C_{t2}$	Costs of false investigations by third party
$C_{g}$	Cost of strict government regulation
$I_{h1}$	Incomes from compliant operation of hospital
$I_{h2}$	Incomes from illegal operation of hospital
$I_{t1}$	Incomes from true investigations by third party
$I_{t2}$	Incomes from false investigations by third party
$I_{g1}$	Social benefits brought to the government by compliant operation of hospital
$I_{g2}$	Social benefits brought to the government by illegal operation of hospital
$A_{h}$	Rewards from local governments for compliant operation of hospital
$P_{h}$	Fines imposed by local government on illegal operation of hospital
$P_{t}$	Fines imposed by local government on false investigations by third parties
$A_{g}$	Rewards from superior government for strict supervision by local government
$P_{g}$	Fines imposed by the superior government on the local government for the loss of social benefits due to non-supervision

**Table 2:** The payoff matrix for the evolutionary game model.

		Third Party	Local Government	
			Strict Supervision (z)	Non-Supervision (1−z)
Hospital	compliant operation (x)	true investigation (y)	$I_{h1}+A_{h}-C_{h1}$, $I_{t1}-C_{t1}$, $I_{g1}-C_{g}-A_{h}+A_{g}$	$I_{h1}-C_{h1}$, $I_{t1}-C_{t1}$, $I_{g1}$
		false investigation (1−y)	$I_{h1}+A_{h}-C_{h1}$, $I_{t1}-C_{t2}-P_{t}$, $I_{g1}-C_{g}-A_{h}+P_{t}+A_{g}$	$I_{h1}-C_{h1}$, $I_{t1}-C_{t2}$, $I_{g1}$
	illegal operation (1−x)	true investigation (y)	$I_{h2}-C_{h2}-P_{h}$, $I_{t1}-C_{t1}$, $I_{g2}-C_{g}+P_{h}+A_{g}$	$I_{h2}-C_{h2}-P_{h}$, $I_{t1}-C_{t1}$, $I_{g2}+P_{h}$
		false investigation (1−y)	$I_{h2}-C_{h2}-P_{h}$, $I_{t2}-C_{t2}-P_{t}$, $I_{g2}-C_{g}+P_{h}+P_{t}+A_{g}$	$I_{h2}-C_{h2}$, $I_{t2}-C_{t2}$, $I_{g2}-P_{g}$

**Table 3:** Eigenvalue of each equilibrium point.

Equilibrium Point	Jacobian Matrix Eigenvalue ($\lambda_{1},\lambda_{2},\lambda_{3}$)	Symbols of Three Eigenvalues	Stability Conclusion	Conditions of ESS
$E_{1}(0,0,0)$	$C_{h2}-C_{h1}+I_{h1}-I_{h2}$, $C_{t2}-C_{t1}+I_{t1}-I_{t2}$, $A_{g}-C_{g}+P_{g}+P_{h}+P_{t}$	(−,−,⊗)	ESS	A
$E_{2}(1,0,0)$	$C_{t2}-C_{t1}$, $A_{g}-A_{h}-C_{g}+P_{t}$, $C_{h1}-C_{h2}-I_{h1}+I_{h2}$	(−,⊗,+)	Saddle point	
$E_{3}(0,1,0)$	$A_{g}-C_{g}$, $C_{t1}-C_{t2}-I_{t1}+I_{t2}$, $C_{h2}-C_{h1}+I_{h1}-I_{h2}+P_{h}$	(−,+,⊗)	Saddle point	
$E_{4}(0,0,1)$	$C_{g}-A_{g}-P_{g}-P_{h}-P_{t}$, $C_{t2}-C_{t1}+I_{t1}-I_{t2}+P_{t}$, $A_{h}-C_{h1}+C_{h2}+I_{h1}-I_{h2}+P_{h}$	(−,⊗,⊗)	ESS	B
$E_{5}(1,1,0)$	$C_{t1}-C_{t2}$, $A_{g}-A_{h}-C_{g}$, $C_{h1}-C_{h2}-I_{h1}+I_{h2}-P_{h}$	(+,−,⊗)	Saddle point	
$E_{6}(1,0,1)$	$A_{h}-A_{g}+C_{g}-P_{t}$, $C_{t2}-C_{t1}+I_{t1}-I_{t2}+P_{t}$, $C_{h1}-A_{h}-C_{h2}-I_{h1}+I_{h2}-P_{h}$	(⊗,−,−)	ESS	C
$E_{7}(0,1,1)$	$C_{g}-A_{g}$, $C_{t1}-C_{t2}-I_{t1}+I_{t2}-P_{t}$, $A_{h}-C_{h1}+C_{h2}+I_{h1}-I_{h2}+P_{h}$	(+,⊗,⊗)	Saddle point or unstable point	
$E_{8}(1,1,1)$	$A_{h}-A_{g}+C_{g}$, $C_{t1}-C_{t2}-I_{t1}+I_{t2}-P_{t}$, $C_{h1}-A_{h}-C_{h2}-I_{h1}+I_{h2}-P_{h}$	(+,⊗,⊗)	Saddle point or unstable point	

Note: ⊗ represents the symbol is unknown. $\begin{array}{l} A:C_{g}>A_{g}+P_{g}+P_{h}+P_{t}\;\\ B:P_{t}<C_{t1}-C_{t2}+I_{t2}-I_{t1};A_{h}+P_{h}<C_{h1}-C_{h2}+I_{h2}-I_{h1}\\ C:A_{h}+C_{g}<A_{g}+P_{t} \end{array}$.

## Data Availability

Not applicable.
